# An Energy Scaled and Expanded Vector-Based Forwarding Scheme for Industrial Underwater Acoustic Sensor Networks with Sink Mobility

**DOI:** 10.3390/s17102251

**Published:** 2017-09-30

**Authors:** Zahid Wadud, Sajjad Hussain, Nadeem Javaid, Safdar Hussain Bouk, Nabil Alrajeh, Mohamad Souheil Alabed, Nadra Guizani

**Affiliations:** 1 Capital University of Science and Technology, Islamabad 44000, Pakistan; zahidmufti@nwfpuet.edu.pk; 2 University of Engineering and Technology, Peshawar 25000, Pakistan; 3 School of Engineering, University of Glasgow, Glasgow G12 8QQ, UK; sajjad.hussain@glasgow.ac.uk; 4 COMSATS Institute of Information Technology, Islamabad 44000, Pakistan; 5 Department of Information and Communication Engineering, DGIST 42988, Korea; bouk@dgist.ac.kr; 6 Biomedical Technology Department College of Applied Medical Sciences, King Saud University, Riyadh 11633, Saudi Arabia; salabed@ksu.edu.sa; 7 Department of Electrical and Computer Engineering, Purdue University, West Lafayette, IN 47907, USA; nguizani@purdue.edu

**Keywords:** Industrial Underwater Acoustic Sensor Networks (IUASNs), energy consumption, energy hole, network lifetime, End-2-End Delay (E2ED), routing

## Abstract

Industrial Underwater Acoustic Sensor Networks (IUASNs) come with intrinsic challenges like long propagation delay, small bandwidth, large energy consumption, three-dimensional deployment, and high deployment and battery replacement cost. Any routing strategy proposed for IUASN must take into account these constraints. The vector based forwarding schemes in literature forward data packets to sink using holding time and location information of the sender, forwarder, and sink nodes. Holding time suppresses data broadcasts; however, it fails to keep energy and delay fairness in the network. To achieve this, we propose an Energy Scaled and Expanded Vector-Based Forwarding (ESEVBF) scheme. ESEVBF uses the residual energy of the node to scale and vector pipeline distance ratio to expand the holding time. Resulting scaled and expanded holding time of all forwarding nodes has a significant difference to avoid multiple forwarding, which reduces energy consumption and energy balancing in the network. If a node has a minimum holding time among its neighbors, it shrinks the holding time and quickly forwards the data packets upstream. The performance of ESEVBF is analyzed through in network scenario with and without node mobility to ensure its effectiveness. Simulation results show that ESEVBF has low energy consumption, reduces forwarded data copies, and less end-to-end delay.

## 1. Introduction

Smart coasts (SCs) are one of the key factors for the sustainable community [1]. The main objectives of the SC are to monitor the quality of water, the ecosystem, water pollution, seismic activity and management of the coastal zone(s). All of these objectives are hard to achieve without continuous detection, collection, monitoring, and management of the oceanographic parameters. This uninterrupted collection and communication of the aquatic parameters are not possible without Underwater Sensor Networks (USN) [2], which is one of the key technologies in the realm of SCs.

USN consists of the battery operated nodes deployed in the aquatic environment, where each node has the capability to sense the physical aquatic and physical parameters, communicate the sensed parameters, and temporarily store a small amount of data due to limited storage. Sensed data is communicated to the Sink(s) that is/are floating at the sea surface that forward(s) data to the onshore monitoring station. Communication in the underwater environment poses many challenges for all the communication approaches that have been considered [3] e.g., radio or electromagnetic [4,5], optical [6,7], and acoustic communication modes [2,8].

Electromagnetic signals at radio frequencies are more suitable for terrestrial communication and offer high bandwidth, low per bit energy, and small propagation delay. Hence, this communication method is enabled over the Sink(s) to communicate with the onshore monitoring station. However, electromagnetic signals at radio frequencies in the underwater environment observe severe attenuation that leads to a very large absorption loss due to high water conductivity. Similarly, the optical signals are not preferable in the aquatic environment because it requires the precise line of sight between sender and receiver, which is quite hard in the presence of the ocean current. Furthermore, the water turbidity severely limits the optical communication distance.

Acoustic communication modality is one of the most widely and preferable for underwater networks, also called underwater acoustic sensor networks (UASNs). It faces challenges such as high error rate due to fading and multipath that limits the available bandwidth [9]. In addition to that, the acoustic speed in an aquatic environment is far less than radio communication in the terrestrial environment. All these factors limit the data routing, synchronization, and maintaining the network state information. Hence, the solutions designed for terrestrial sensor networks can not directly be applied to UASNs.

Long propagation delay, high energy consumption, low bandwidth, and large error rate are the salient and intrinsic characteristics as well as the challenges of acoustic communication in the underwater networks. Most of these challenges are correlated with each other. Most of the vector based routing schemes proposed for the UASN employ holding time that is distributively computed by each node using the local node or network parameters, e.g., distance to sink, proximity to the center of the virtual pipeline between the sender and sink, distance to the previous hop sender and the receiving node, etc. First, nodes check either they are within the virtual pipeline or not. Once a node ensures that it is located within the virtual pipeline, then it estimates the holding time. Holding time is estimated every time when a node receives the first copy of the packet from downstream nodes. The timer is triggered and its duration is set to the estimated holding time period. Once the timer expires and if that node does not receive any other copy from its neighbors, it will forward the packet and all the other nodes will suppress the packet forwarding. A preferable forwarder must have the smallest holding time compared to its immediate neighbors and is desirable to forward the packet.

For example, to avoid long propagation delays, vector based routing schemes consider node’s proximity information between the sender and the sink node, and nearness to the pipeline center in the holding time. It is projected that the packet forwarding through these nodes reduces the end-to-end delay. However, all communication through these nodes will deplete their energy and result in a void energy hole problem. Therefore, the energy fairness should be achieved among all the nodes within the vector as well as in the network. Hence, the energy factor must be considered in the holding time computation to increase network lifetime. Nevertheless, the nodes with sufficient energy do not guarantee the shortest path (with small end-to-end delay) between sender and sink. On the other hand, better forwarding decisions or precise holding time estimation can be attained if an updated network state (complete or partial network) information is available at each node in the network. This network state information availability is possible through the exchange of control packets, which again impacts the bandwidth, energy, and inflates the error rate. Hence, any forwarding scheme for UASN must consider the constraints and provide the mediated solution. Additionally, the difference between holding times estimated by all the immediate neighbors should be larger than the propagation delay between them to properly suppress the unnecessary packet forwarding. Otherwise, many copies of the same packet will be forwarded in the network.

It is a well established fact that the acoustic signal consumes more energy and experiences a very long propagation delay and channel error in the underwater environment [10]. The propagation delay and energy consumption increases drastically if the farthest acoustic node in the network needs to communicate data towards the sink that placed at the fixed location. In order to efficiently collect data, different schemes in literature adopt sink mobility. Mobile sink, also called mobile station, can be any node that moves in the aquatic environment either autonomously, e.g., autonomous underwater vehicle (AUV) [11], over the anchored rope, vessel, etc. The mobile sink is considered to have sufficient available resources to roam in the network (frequent refueling and/or recharging). Hence, any routing scheme proposed for the UASN must also be analyzed in the mobile sink UASN scenario to verify its effectiveness.

The organization of the remaining manuscript is as follows: Previous work related to the proposed ESEVBF is reviewed in Section 2. In Section 3, holding time and the working principle of ESEVBF is described. Simulation analysis in terms of energy consumption, end-to-end delay, the number of copies of data packets, packet delivery ratio, and average hop count, in the underwater network without and with sink mobility, is performed in Section 4. Finally, the discussion is concluded in Section 5.

## 2. Previous Work

In the realm of UASNs, there is a plethora of research to achieve efficient routing in the network [12,13,14]. However, here we will only focus on the specific domain of routing protocols that are related to our proposed scheme. Thus, the previous works that suppress the packet broadcast in underwater acoustic sensor networks using node location information [15] and the holding time [16], are discussed in this section.

Vector based forwarding (VBF) has been proposed in [17]. VBF considers the node’s location information and forwards packets through all the intermediate nodes that lie in the virtual pipeline between source and destination node pairs. When a node receives a packet from the downstream node, it first checks whether it is within the virtual pipeline or not. If a node is within the virtual pipeline, then it computes holding time using the *desirableness factor* of the forwarder, α, maximum predefined delay, and the propagation delay towards the edge of the transmission range of the sender node. A desirableness factor includes the ratio of the node’s distance from the center of virtual pipe and width of the virtual pipeline plus the distance from the sender node. Every time when the same sender forwards the packet, the particular set of node(s) in the neighborhood closer to the center of the virtual pipeline are penalized and die out earlier. The number of redundant packet transmissions increases when network density increases because the delay difference between their holding time becomes negligible. Accordingly, excessive energy is consumed and more packets fail to reach the Sink node. The radius of the pipeline is not fixed in the VBF. Finally, in a sparse network scenario, it is really hard to find nodes within the virtual pipeline between sender and the sink node pairs. In other words, there must exist a single path inside the virtual pipeline between sender and sink to successfully forward packet towards the sink, which is hard in the sparse network scenario.

Instead of using a single virtual pipeline between sender and the Sink node, authors proposed hop-by-hop VBF (HH-VBF) [18] that forms a separate pipeline between the Sink and each forwarding or relaying hop. The authors assume that is better to form a hop-by-hop relative pipeline to find more suitable packet forwarders. The radius of the pipeline is similar to the transmission range of the node. The holding time computation in HH-VBF is not different than the VBF. HH-VBF fairly improves the packet delivery ratio compared to VBF because it increases the chance of finding more suitable forwarder within the hop-by-hop virtual pipeline. As VBF, HH-VBF fails to provide energy fairness within the network. In addition to that, HH-VBF fails to avoid any void region in the pipeline path between source and the sink node.

The authors in [19], proposed Adaptive HH-VBF (AHH-VBF). It is claimed that AHH-VBF adaptively adjusts forwarding distance and the transmission power. The forwarding distance regulated base on the 1-hop neighbor density at each hop and the transmission power is computed to the maximum distant forwarder in the range. The radius of the hop-by-hop pipeline is controlled to reduce the packet forwarding by many nodes in the forwarding region. In order to achieve transmission power and forwarding area adaptiveness, AHH-VBF sends multiple *Request* messages at different power levels and maintains the neighborhood table when it receives Acknowledgement packets in response to the *Request*. If the number of neighbors found during this process is less than τ, then transmission power is set to the maximum power; otherwise, it is adjusted accordingly. The energy efficiency is achieved through the transmission power adjustment and pipeline radius. However, several data packet transmissions from the same source node will always select the same set of forwarders, which violates the energy fairness in the network as in HH-VBF. Additionally, the power adaptiveness does not guarantee that avoidance of packet duplication and as well as the potential forwarder selection.

In [20], authors proposed two routing schemes named as avoiding void node with adaptive hop-by-hop vector based forwarding (AVN-AHH-VBF) and cooperation-based AVN-AHH-VBF (CoAVN-AHH-VBF). The AVN-AHH-VBF selects the next hop forwarder based on the neighborhood information. The main selection criterion of the next hop forwarder is the node with maximum number of neighbors. It means that all the network nodes share their 1-hop proximity information as well using the neighbor request and neighbor acknowledgment packets. The holding time to suppress duplicate data broadcast in the pipeline is similar to AHH-VBF with only one additional parameter, that is the number of neighbors. On the other hand, the CoAVN-AHH-VBF works in a sender initiated manner, where it forwards the next hop forwarder(s) information in the data packet. The nodes that receive the data packet first search their ID in the packet and once they find their ID, they participate in the forwarding process. In addition to the next hop forwarder in the CoAVN-AHH-VBF checks whether the Bit Error Rate (BER) to the sender node is greater than the threshold or not. If BER is greater than the threshold then retransmission request packet is sent by the forwarder to the sender node to send data packet again. Both the schemes have large control overhead and incur more delay due to control packet exchange and practically they are not suitable for the underwater acoustic network scenario.

Next, the authors have proposed sender initiated void avoidance routing protocols named two-hop adaptive hop by hop vector-based forwarding (2hop-AHH-VBF) and quality forwarding adaptive hop by hop vector-based forwarding (QF-AHH-VBF) in [21,22]. These protocols two hop neighborhood information to forward data packets towards the sink(s). The authors compute the composite function involving the energy and distance information of the potential forwarders in the virtual vector. In addition to that, the authors have also used the holding time to avoid duplicate data packet transmission in the virtual pipeline proximity. These schemes also face the same set of problems and face the same shortcomings as in [20].

For this purpose, minimization of void hole probability particularly in local sparse regions is focused on in this paper. The two-hop adaptive hop by hop vector-based forwarding (2hop-AHH-VBF) protocol aims to avoid the void hole with the help of two-hop neighbor node information. The other protocol, quality forwarding adaptive hop by hop vector-based forwarding (QF-AHH-VBF), selects an optimal forwarder based on the composite priority function. QF-AHH-VBF improves network good-put because of optimal forwarder selection. QF-AHH-VBF aims to reduce void hole probability by optimally selecting next hop forwarders.

Following is the discussion about the location based routing protocols for underwater networks that do not consider any holding time.

The concept of directional power adaptiveness to overcome the packet flooding in underwater networks is also proposed in the Focused Bream Routing (FBR) [23]. Power and flooding angle are gradually increased according to the predefined gradients before forwarding data packets. A node requires to send many Request to Send (RTS) packets and waits for the Clear to Send (CTS) packets from neighbors in the beam direction. The nodes also share their location information in the RTS-CTS exchange. In order to confine the flooding direction, FBR selects potential node(s) in the beam direction that are eligible to forward the data packets.The power level is controlled by the FBR based upon the suitable number of neighbors located during the RTS-CTS exchange between the source and its neighboring nodes. The main drawback of the FBR is that it has a very large delay and energy consumption because of the initial RTS-CTS packet exchange. In case of the sparse network, the gradual power level increase will pose unnecessary communication delay.

FBR faces RTS and CTS delay, which is reduced by scheme named Layer by Layer Angle Based Flooding (L2-ABF) [24]. In L2-ABF, the flooding angle of the data packet in a cone shape towards the upper layer (towards the direction of Sink node) is directly adjusted without exchanging RTS-CTS. The power and angle (the length and width of the cone) depend on the layer distance and relative node speed between sender and the receiving nodes, respectively. The protocol minimizes the flooding of the data packets, however, it consumes more energy in the sparse networks because of many attempts to send data in the sparse network with no forwarding candidate in the cone. Additionally, it is not an energy efficient in the dense network environment as well because multiple copies of data packet may be forwarded by different nodes in the cone. The L2-ABF does not use any mechanism to avoid void hole in the underwater environment.

In [25], proposed the state-less, location- and receiver-based routing protocol named Directional Flooding-based Routing (DFR). All nodes in DFR knows their own location, location of sink node, and the location their immediate neighbors. DFR does not employ any holding time, which means that all the nodes that receive the copy of a data message will further forward towards upstream. However, DFR controls the flooding direction of the data packets within the certain zone in the direction of Sink node. Size of the flooding zone is adapted with the upstream link quality. As link quality fluctuates in the underwater environment, the flooding zone unnecessarily becomes wider that consumes more energy and reduces delivery ratio. In addition that, void region can still appear in the flooding area and it worsens when the flooding zone decreases due to increase in the link quality of neighboring nodes. However, DFR shrinks the flooding zone upto a point that there should be at least one forwarder in the zone. This can not guarantee the void avoidance in the forwarding zone.

In literature, there are different proposals that solely focus on the *sink mobility* and use depth information to select next hop forwarders to minimize the end-to-end delay, reduce data collection energy cost, and increase packet deliver ratio. Following is the brief discussion of the sink mobility based data collection schemes.

Authors in [26], proposed an AUV (which acts as a mobile sink) based distributed data-gathering scheme to efficiently collect data from the selected nodes, called path-nodes, instead of traversing the whole network. The path nodes are the data collection points and are optimally selected to shorten the AUV trajectory as well as achieve network energy efficiency. These nodes are cluster heads and selected during the clustering phase and then they collect data from the member nodes. The AUV collects the data from selected nodes in the trajectory and saves the network energy. However, the cluster formation and deformation pose additional overhead and the communication delay becomes higher if the selected clusters are at long distances.

Authors in [27], proposed the AUV-aided underwater routing protocol (AURP) that collects data from multiple points in the network, called gateway nodes. AURP uses controlled mobility to minimize the AUV’s mobility path and collects data through heterogeneous acoustic communication channels. This heterogeneous channel enables AUVs to select either short-range high data rate or long-range low data rate channel. AURP saves network energy, however, it fails to minimize energy consumption and data forwarding delay in situation where many nodes are associated to a single gateway and the sparse network scenario, respectively. A mobile geocast or mobicast in the three-dimensional (3D) AUSN with mobile sink has been investigate in [28]. The main objective of the mobicast is to minimize energy consumption and avoid energy hole problem during data collection. The whole 3D UASN is divided into multipe 3D geographic zones that are also called zone of reference (ZOR). The AUV collects data from the sensor within the ZOR and moves through the user-defined path. The sensors within the ZOR conserve their energy by only waking up at the AUV’s visit time.

In [29], the authors proposed the ESDR (Event Segregation-based Delay sensitive Routing) and the forwarding process depends upon the criticality level of the sensing event. The data related to the very critical and the critical events is forwarded directly to the base station due to their delay sensitive requirements. However, the normal nodes that generate normal event data with less priority and not delay bound, forward their data packets towards the mobile sinks, which are AUVs and referred as courier nodes (CNs). The delay bound data is forwarded using the forwarding functions that consider the cross-layer parameters including the Localization Free SNR (LFSNR), Energy Cost Function (ECF), Depth-Dependent function (DDF), and Signal Quality Index(SQI). The estimation of those functions is quite complex as well as the end-to-end delay for general category data is very high in the ESDR. In addition to that, the author used the constant depth threshold to restrict the CN’s mobility, which is quite difficult to achieve in generic underwater acoustic network scenario. The holding time to guarantee the packet suppression considers the constant value and the estimation of that constant value is impractical in the unpredictable aquatic environment. The sink mobility based schemes are best summarized in the survey in [30].

S. M. Ghoreyshi et al. in [31] proposed Energy-efficient and Void Avoidance Depth Based Routing (EVA-DBR) protocol. Authors claimed that the EVA-DBR is the stateless routing protocol, however, EVA-DBR updates the neighboring node table that is used to select next hop forwarders. The neighborhood table is maintained by periodically exchanging the control packets. Based on the information in the neighboring table, the size of the forwarding zone is adopted. The authors used different thresholds and forwarding ranges, if the number of nodes with lower depth in that forwarding range are greater than the threshold, then minimum forwarding range is used in the data packet. On the contrary, the forwarding range is increased and matched with the threshold until the maximum forwarding range reaches. EVA-DBR minimizes the energy consumption, however, the control packet cost is very high in the dense network and duplicate data packet suppression holding time used does not guarantee the packet suppression.

From the above mobile sink based literature review, we have observed that the sink mobility improves the network efficiency in terms of battery power, packet delivery ratio, and so on. Hence, any scheme proposed for the UASN must be tested with and without sink mobility to verify its effectiveness.

### Motivation and Contributions

Most of the vector based forwarding schemes are merely focused on the packet forwarding by estimating the holding time using the node’s directional and position information. Energy efficiency is achieved through transmission power adaptation but not the available energy of the nodes. The proximity closeness of the node towards the sink minimizes the end-to-end delay. Inspired from the above discussion, we proposed a novel energy scaled and expanded vector based (ESEVBF) for UASNs. ESEVBG estimates holding time of the potential forwarders by keeping the following points under consideration.

1. The holding time of all the potential forwarders is scaled using the neighboring nodes’ energy information. It increases the holding time *difference* between them even for a small variation in the energy level of neighbors.
2. The expanded proximity closeness ratio of the forwarding candidate nodes towards the virtual pipeline between sender and sink is added in holding time computation to signify the node preference.
  Both (1) and (2) scale and signify the holding time difference between the candidate forwarders for small parameter variance. This ensures that all nodes in the transmission range of the suitable forwarder (with minimum holding time) must receive copy of the packet before their holding time expiration.
3. Each candidate forwarder uses its neighboring node information to find suitability to abbreviate its holding time duration to curtail the end-to-end delay.
4. Energy efficiency and energy balancing are achieved by employing the normalized residual energy information of the neighboring nodes in the holding time and suppressing more number of packets.
5. No constant parameters in the holding time estimation are used.
6. The proposed scheme is analyzed in the network scenarios with and without sink mobility.

The simulation results show that ESEVBF improves energy efficiency and reduces end-to-end delay without compromising the reliability compared to its counterpart, AHH-VBF.

## 3. Proposed Scheme

In this section, we present the detailed discussion of our proposed scheme. Our scheme is compared with the AHH-VBF that uses the holding time $HT_{p}^{i}$ of node *i* to forward packet *p* towards the Sink *D*. The $HT_{p}^{i}$ suppresses extra copies of *p* by selecting the potential forwarder *i* using its projection distance from the center of the virtual cylinder or pipeline, distance towards *D*, and distance from the node *S* (a node from which *i* received a copy of *p*). The terms virtual pipeline or virtual cylinder are interchangeably used in the context of this paper. AHH-VBF adaptively adjusts the transmission power and radius of the virtual cylinder to its maximum distance mobile neighbor. In contrast to AHH-VBF, the proposed scheme estimates $HT_{p}^{i}$ based on the normalized residual energy scaled distance from *S*, expanded distance from the virtual cylinder’s centerline, and distance towards *D*. The resultant $HT_{p}^{i}$ prioritize the nodes that have large residual energy, near the center of the virtual cylinder, and least distant to *D*. In addition to that, it also increases the difference between holding times of all nodes in the potential forwarding zone to suppress more copies of *p*. In result, the packet collision at next hops can be avoided and network energy can be conserved to maximize the network lifetime.

### 3.1. Problem Statement

When a node *S* transmits the data packet (either that data packet is generated by that node or received from the downstream sensor nodes), all the neighboring nodes within its $T_{r}^{S}$ and in the *PFZ*, receive that packet. Now, the question arises that which node(s) has(ve) to further transmit or relay the packet in upstream direction? The answer to this question is the holding time, HT. Upon successful reception of packet *p*, a node *i* computes the $HT_{p}^{i}$ and starts the timer. During $HT_{p}^{i}$ is on, *i* does not forward the packet. However, node *i* can receive data packets from its neighboring nodes, which may be copies of *p* or other data packets. When node *i* receives additional one or more than one copies of *p* while $HT_{p}^{i}$ did not expire, then it suppresses the transmission of *p*. On the contrary, if $HT_{p}^{i}$ expires and no copies of packet *p* have been received during the $HT_{p}^{i}$ period, then *i* forwards the packet *p*. This simple phenomenon alleviates the extra broadcast overhead, which is necessary for the UASN scenario where energy and bandwidth are the scarce resources. However, UASN has an added feature that must be considered while designing the holding time, which is the long propagation delay.

**Figure 1:**
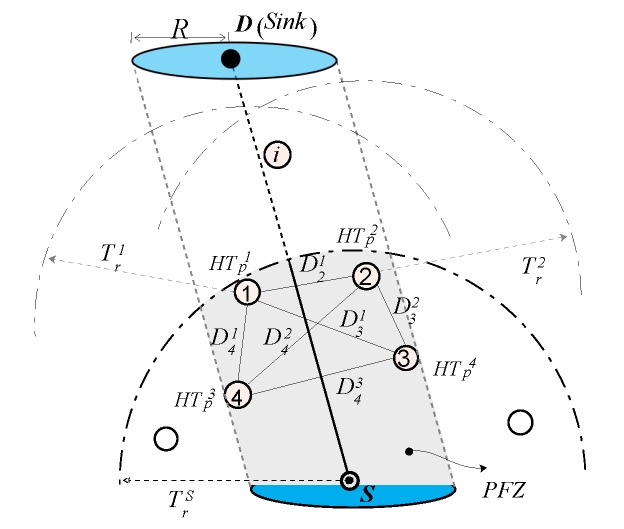
Holding Time and PFZ scenario.

Consider a scenario where multiple nodes in the *PFZ* receive *p*, then all nodes in *PFZ* will calculate their respective holding time $HT_{p}^{i}$. If the number of nodes in PFZ>1, then the difference between their holding times must be greater than the propagation delay between them. Let, holding time of nodes 1 and 2 in *PFZ* for data packet *p* is $HT_{p}^{1}=1.2$ *s* and $HT_{p}^{2}=1.3$ *s*, refer Figure 1. And let the propagation delay between both the nodes 1 and 2 is $\frac{D_{2}^{1}}{v_{s}}=0.2$ *s*, where $v_{s}$ is the acoustic signal speed in the aquatic environment. In this scenario, node 1 will forward the packet after 1.2 s, however, due to a long propagation delay and the short holding time difference, 2 will not receive the copy of *p* from node 1 and its $HT_{p}^{2}$ will expire. Hence, 2 will also forward the packet *p*. Similarly, any other node(s) in the *PFZ* that has/ve holding time difference less than the propagation delay between them, will also forward(s) the packet *p*. Therefore, even by applying the holding time, multiple copies of the same packet will be forwarded by the nodes in the PFZ that will impact the energy consumption as well as the packet collision at the next hop receiving nodes, e.g., $n_{i}$ in the network scenario shown in Figure 1.

**Figure 2:**
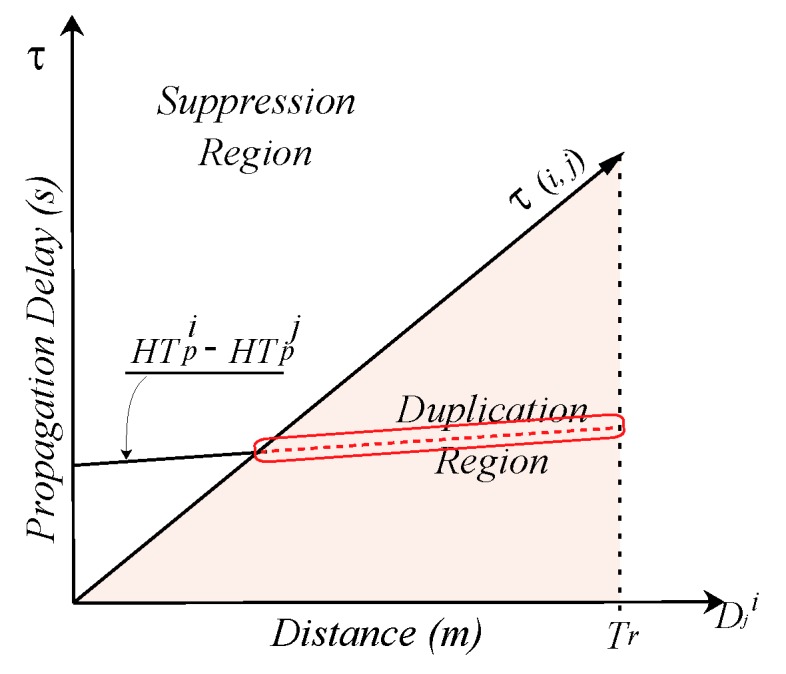
Relationship between holding time difference and broadcast suppression in the underwater networks.

From the above discussion, it is observed that there is a close relationship between the holding time difference between the close proximity neighbors, especially in the underwater communication scenario. This relationship is shown Figure 2. A well-established fact about the underwater acoustic networks is its long propagation delay that is one of its limitations to be considered by any packet forward scheme. The figure also shows that the propagation delay between node *i* and *j*, $\tau_{\left(i,j\right)}$, that is directly proportional to the distance between them. It is obvious that if the difference between the holding time of node *i* and *j* for packet *p*, $HT_{p}^{i}$-$HT_{p}^{j}$, is greater than the $\tau_{\left(i,j\right)}$, then the packet suppression can be achieved. Otherwise, if the $HT_{p}^{i}$-$HT_{p}^{j}<\tau_{\left(i,j\right)}$, then multiple copies of *p* will be broadcasted in the network. The shaded area in the figure is the duplication zone. This can easily be avoided when the holding time difference is larger than the propagation delay. One of the drawbacks of the larger holding time is the long end-to-end delay that should be avoided in holding time-based forwarding schemes.

Based on the above discussion, we proposed the new packet forwarding scheme that suppresses the data packet broadcast storm by adapting the novel energy scaled and expanded holding time estimation and neighbor information based data forwarding in the underwater acoustic networks. A detailed discussion about the proposed holding time computation and the forwarding schemes is discussed below.

### 3.2. Preliminaries

Following is the brief description of the notations that have been used in our proposed forwarder selection scheme.

- *Neighbors of node i, ($\xi_{i}$)*: All the nodes that are in $T_{r}^{i}$ form which *i*.
  (1) $$\xi_{i}=|\left\{j\in N|D_{j}^{i}\leq T_{r}^{i}\right\}|$$
  where N is the set of nodes in the network and $D_{j}^{i}$ is the euclidean distance between *i* and *j* in three-dimensional euclidean space:
  (2) $$D_{j}^{i}=\sqrt{{\left(x_{i}-x_{j}\right)}^{2}+{\left(y_{i}-y_{j}\right)}^{2}+{\left(z_{i}-z_{j}\right)}^{2}}$$
- *Potential Forwarding Zone (PFZ)*: PFZ is the region of between node $S(x_{S},y_{S},z_{S})$ (that currently forwarded the packet *p*) and Sink $D(x_{D},y_{D},z_{D})$. PFZ is the subregion of $T_{r}^{S}$ of node *S* and the nodes in the region are called potential forwarder nodes (PFNs), which are preferable to further relay *p*. Any point in 3D euclidean space $f(x_{f},y_{f},z_{f})$ is considered to be in the PFZ of *S*, if it satisfies the following conditions:
  $$\begin{array}{cc} D_{D}^{f} & <D_{D}^{S},\\ D_{S}^{f} & <T_{r}^{S},\;\mathrm{and}\\ z_{f} & \leq z_{S}. \end{array}$$
  Neighbors of node *i* that are in PFZ of *S*:
  (3) $$\chi_{i}=\left\{n_{i}\in \xi_{i}|D_{n_{i}}^{i}\leq T_{r}^{i}\wedge D_{S}^{n_{i}}\leq T_{r}^{S}\wedge z_{n_{i}}\leq z_{S}\right\}$$

### 3.3. Estimation of $HT_{p}^{i}$

Every node *i* that is within the *PFZ* first computes its holding time $HT_{p}^{i}$, when it receives the packet *p* from *S* as follows:

(4) $$HT_{p}^{i}=\alpha +\beta +\overset{\gamma }{\overset{︷}{1-\left(\frac{D_{D}^{S}-D_{D}^{i}\cos\left(\theta_{i}\right)}{T_{r}^{S}}\right)}}$$

The first factor of HT expression, α, considers the distance of potential forwarder from the edge of the $T_{r}^{S}$ that is scaled with the inverse normalized residual energy of the node. Any node that is closest to the edge of the $T_{r}^{S}$ and has the maximum residual energy will be logically preferable forwarder and α is computed as:

(5) $$\begin{array}{cc} \alpha & =e^{\left(-E_{i}\right)}\left(\frac{T_{r}^{S}-D_{S}^{i}}{v_{s}}\right) \end{array}$$

where

$$\begin{array}{cc} E_{i} & =\frac{e_{i}-e_{min}}{e_{max}-e_{min}}\\ e_{min} & =min\left(e_{j}|\forall j\in \chi_{i}\right)\\ e_{max} & =max\left(e_{j}|\forall j\in \chi_{i}\right)\\ E_{i} & \in [0,1] \end{array}$$

The energy of a node is relatively normalized to all the neighboring nodes’ residual energy in that are neighbors of *i* and in PFZ. The node with maximum residual energy within the neighborhood, including the current forwarding node, will have the $E_{i}=1$ and vice versa. In AHH-VBF, this factor increases the chances of node *i* to become potential forwarder if it is at the edge of the of $T_{r}^{S}$. On the contrary, the proposed scheme scales this parameter using the scaled residual energy of node *i*. The $e^{\left(-E_{i}\right)}$ element in α decreases overall $HT_{p}^{i}$ of node *i* with larger residual energy and makes it more suitable candidate to forward *p*.

**Figure 3:**
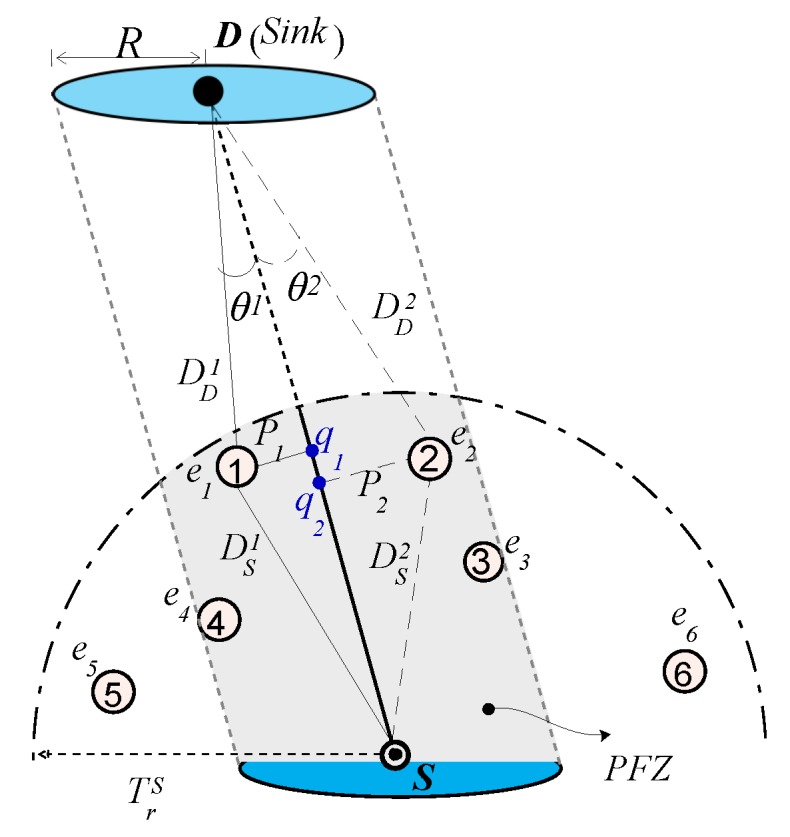
Holding time estimation parameters and scenario.

The next factor of the HT, β, is the ratio of the projection distance $P_{i}$ of the potential forwarding node *i* from the centerline of the virtual cylinder with radius *R*. This centerline connects nodes *S* and *D* that are at the center of the lower and upper faces of the cylinder. Nodes that are furthest from this centerline are not desirable as forwarders and their HT must be larger than the one that are closer the centerline, as shown in Figure 3. To achieve this, AHH-VBF just takes the ratio of $P_{i}$ and *R*, $\beta =\frac{P_{i}}{R}$, which returns the value of β within the closed interval [0,1]. However, the value of β should be expansed to widen this value to easily avoid multiple data transmissions as:

(6) $$\beta =\tan\left(\frac{P_{i}}{R}\right)$$

where $P_{i}$ is estimated as:

$$\begin{array}{cc} P_{i} & =\left(2\times A\right)/D_{D}^{S},\\ A & =\sqrt{\rho \times \left(\rho -D_{D}^{S}\right)\times \left(\rho -D_{D}^{i}\right)\times \left(\rho -D_{S}^{i}\right)},\;\mathrm{and}\\ \rho & =\left(\frac{D_{D}^{S}+D_{D}^{i}+D_{S}^{i}}{2}\right).\; \end{array}$$

The last factor of the HT, γ, projects the distance of the potential forwarder towards the sink. Any node in *PFZ* that is closer to the sink is a suitable to be the next potential forwarder. The γ of all the nodes in *PFZ* is between [0, 1]. In this factor, the element $D_{D}^{i}\cos\left(\theta_{i}\right)$ results the distance between the projection point $q_{i}$ on the centerline and the Sink node. Here, $\theta_{i}$ is calculated as:

(7) $$\theta_{i}=cos^{-1}\left(\frac{{D_{D}^{S}}^{2}+{D_{D}^{i}}^{2}+{D_{S}^{i}}^{2}}{2\times {D_{D}^{S}}^{2}\times D_{D}^{i}}\right)$$

The ratio of the difference between $D_{D}^{S}$ and $D_{D}^{i}\cos\left(\theta_{i}\right)$ and $T_{r}^{S}$ will be high when node *i* is closer to the sink and vice versa. Subtraction of that ratio from 1 will have a very small increment in the holding time of node *i* if it is closer to the sink node, enables node *i* to be more suitable forwarding candidate. On the hand, the holding time of node *i* will be sufficiently increased when it is far from the sink and closer to *S*. In order to efficiently forward the data packet, multiple packets are exchanged between nodes to maintain 1-hop neighboring state at each node. These packets include neighbor request (**NEIGH_REQ**), neighbor acknowledgment (**NEIGH_ACK**), and **data** packet. The structure, header format, and the purpose of all those packets is similar to the one that is used in [19]. Similarly, the same set of steps are followed by our proposed scheme when it receives **NEIGH_REQ** and **NEIGH_ACK**. Because the prime objective of our proposed scheme is to select more suitable data packet forwarders, hence, we proposed a new set of steps when **data** packet is received by node *i*. Detailed working principle of our proposed **data** packet forwarding algorithm is shown in Algorithm 1.

**Algorithm 1:** Proposed data Packet Forwarding Algorithm.
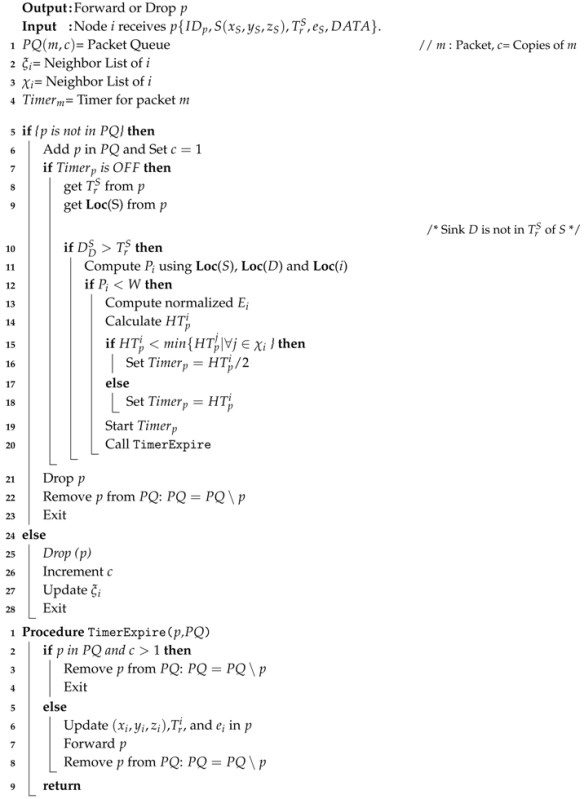


When node *i* receives data packet *p*, it first checks, whether the packet is already in the Packet Queue (PQ), waiting to be forwarded or not. In case of no packet found in PQ, timer for that packet is not active, and *D* is in $T_{r}^{S}$, then *i* checks its position that either *i* is PFZ or not. Accordingly, node *i* computes its $HT_{p}^{i}$ and initiates the timer $Timer_{p}$. During the $Timer_{p}$ is active, node may receive multiple copies of *p* form other nodes and records the number of copies received. Once the $Timer_{p}$ expires, node *i* forwards the **data** packet if it received only single copy of the packet, otherwise, it drops and removes the packet from PQ.

To conclude this subsection, we have proposed the holding time that uses an energy scaled closeness to the edge of the $T_{r}^{S}$, expanded proximity to the centerline of the cylinder between *S* and *D*, and adjacency towards the sink node. Collective, all those factors are necessary for the selection of the appropriate forwarder.

## 4. Simulation Analysis

In this section, the detailed simulation analysis of the proposed scheme in contrast to the conventional AHH-VBR scheme is presented. To fairly evaluate the performance of both the schemes, we simulated an underwater 3D network of 2 km × 2 km × 4 km area, where $X_{max}=Y_{max}=$ 2 km and depth of $Z_{max}=$ 4 km. The network size of 200 to 450 nodes has been simulated with the varying transmission ranges, ranging from 500 m to 900 m to demonstrate the sparse and dense network scenarios. In each simulation trial, the nodes are deployed randomly in the said network area and every individual sensor node acts as a data source (generates data packets) as well as a forwarder node. The position of the Sink node is static during the whole simulation course. Sink node is positioned at the water surface and at the center of the network area with coordinates ($X_{max}/2$, $Y_{max}/2$, 0). All nodes are homogeneous in terms of transmission range in every trial and are assigned initial energy $E_{0}$ as $E_{min}+rand\left(E_{rand}\right)$, where $E_{min}=50j$ and $E_{rand}=30j$. A single network scenario for a given transmission range and network size is simulated 100 times. Therefore, all distinct points in the graphs of the simulation results are an average of 100 simulation trials.

The payload size of the data packet, neighbor request, and acknowledgment packets are 70×8 bits, 64 bits, and 112 bits, respectively. The common header of 88 bits is used for all packet types in simulation. In addition to that, the data rate of $16\times 10^{3}$ bits per second and the underwater acoustic delay propagation delay of 1500 m has been set in the simulations. Network is static during the complete simulation period. In last, the pure ALOHA is used at MAC layer because it is not susceptible to delays and does not use any collision detection and the avoidance mechanism.

As stated earlier in a brief discussion about the conventional AHH-VBF, it ensures the packet forwarding reliability by setting the minimum forwarder threshold, τ, which depends upon the error probability, the packet collision rate, and the size of the packet. However, in simulations, AHH-VBF considered τ≥2, which indicates that there should be at least two or more than two forwarders in the forwarding region. This ensures the reliability as well as the collision probability at the next hop forwarder(s). Additionally, it consumes more energy and utilizes more bandwidth, which is the scarce resource of the UASN. This situation can easily arise when the holding time difference between two or more than two forwarders is very negligible or smaller than the propagation delay between them. On the contrary, our proposed scheme intends to avoid multiple transmissions of the data message towards the upstream direction, to save energy and avoid collision at upstream receivers. In short, the proposed scheme not only selects the spatially suitable but also the energy-rich acoustic node among the forwarders pool. Hence, the fair performance comparison is achieved by setting τ=1 and analyzing the packet suppression count or the number of forwarders count and energy consumption. During all simulation scenarios, there are 200 data sources that generate data packets destined to Sink node. The same number of data sources is used for the large network size scenarios with the intention to find the impact of identical data traffic on network performance.

**Figure 4:**
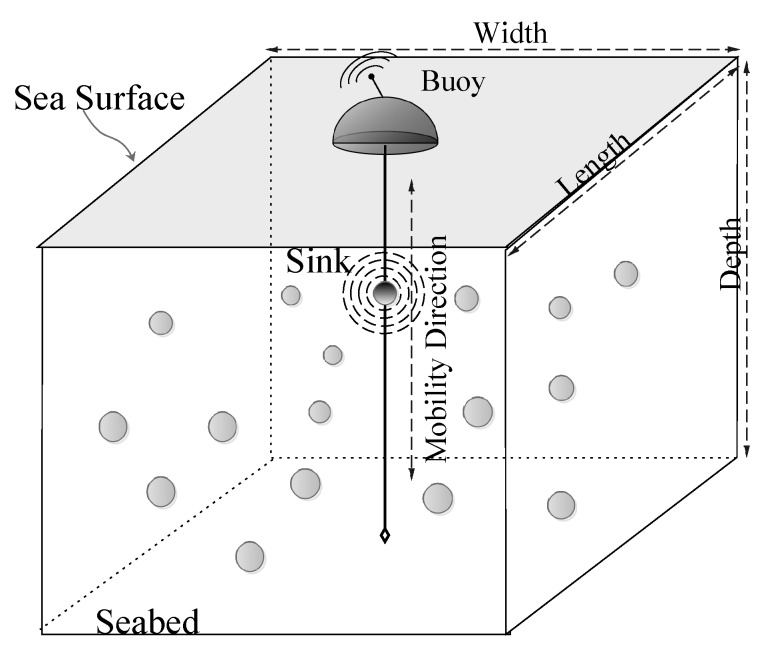
Mobile sink network scenario.

The simulation results are analyzed for two underwater network scenarios: One with the static Sink, which is placed on the sea surface at the fixed location and the other where the Sink is mobile. In the static Sink scenario, the Sink is placed at ($X_{max}/2$, $Y_{max}/2$, Z=0) coordinates. On the contrary, in the mobile Sink network scenario, the sink moves vertically from the sea surface towards the seabed with a constant speed, sp= 5 m/s. However, its *X* and *Y* coordinates remain constant. Once the sink reaches the seabed, it floats upward towards the sea surface with the same speed. Example sink mobility scenario is shown in Figure 4, where sink moves vertically through the cable holding the anchored surface buoy. The primary objective of considering the Sink mobility scenario is the test performance of the proposed scheme in diversified network paradigms.

### 4.1. Performance Metrics

Following is the brief description of the performance metrics that are analyzed through simulations.

- *Total forwarded copies of data*: represents the number of copies forwarded in the network for all data packet transmissions initiated by the source nodes.
- *Number of dead nodes*: is the total number of nodes that could not participate in the data forwarding process because they have residual energy less than the transmission energy.
- *End-to-end delay*: is the cumulative delay experienced by the data packet between its source and the sink node.
- *PDR (Packet Delivery Ratio)*: is the ratio of successfully received data packets by sink over the total number of generated data packets.
- *Energy consumption*: total energy consumption in the network during the whole simulation time.
- *Hop count*: represents the average number of hops that the data packets have traversed between source and sink node.

Following is the brief discussion about each performance metric that is estimated through simulations in the static Sink network scenario.

### 4.2. Simulation Results in the Static Sink Scenario

In this section, all the results are estimated for the network scenario with static sink. In this case, the Sink is placed at sea surface and the center of the network deployment region.

**Figure 5:**
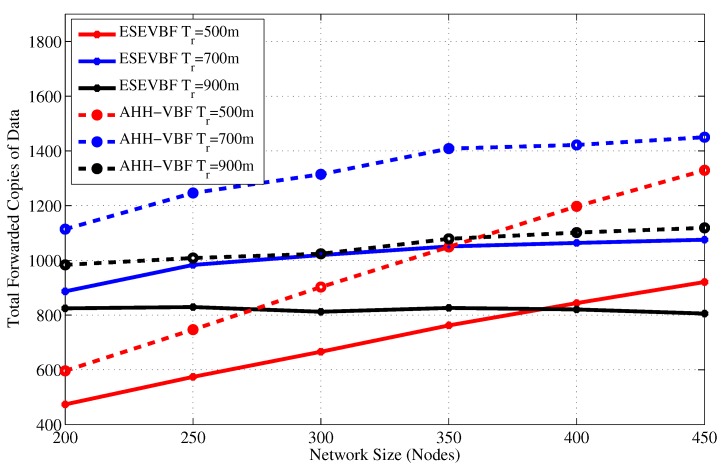
Number of data message copies forwarded in the network for different network size.

**Figure 6:**
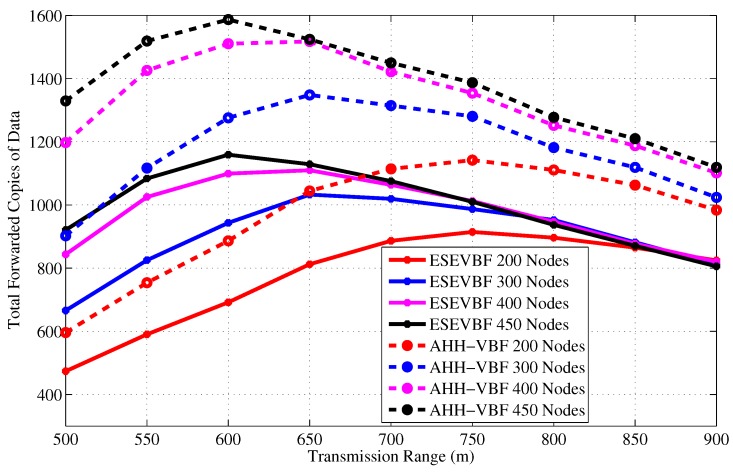
Number of data message copies forwarded in the network for different transmission range.

Figure 5 and Figure 6 show the total number of copies of the data message forwarded in the network versus verying network size and transmission range, respectively. It can be seen in Figure 5 that in a sparse network scenario, $T_{r}=500$ and network size, the total copies of data packet forwarded in the network is smaller than the dense network scenario. The reason behind this phenomenon is that most of the copies of the data packet fail to reach the next hop forwarders. As the network size increases, more copies of the data message are successfully propagated in the network. On the contrary, for large transmission range and small network size, more data packets are successfully forwarded in the network, but when the network size increases, most of the packets are dropped in the network due to a large collision probability. Increase in data packet copies due to large transmission range plus network size is due to two factors; (a) increase in the pipeline radius directly surges in the number of forwarding candidates in PFZ, (b) the propagation delay between potential forwarders in PFZ will be longer and could be more than the holding time difference between them.

**Figure 7:**
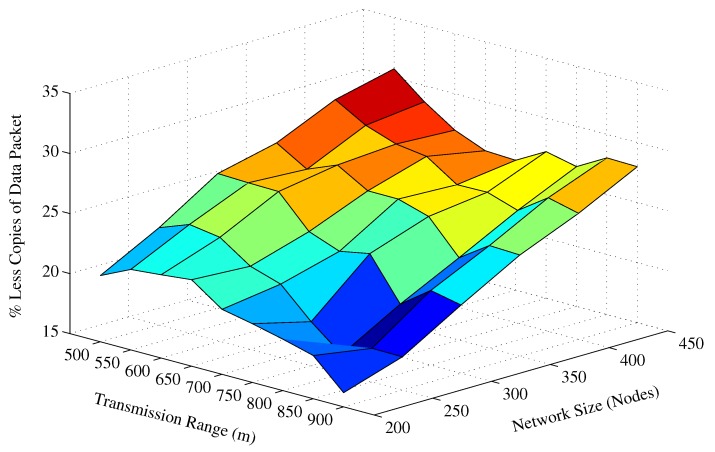
Overall data packet suppression achieved by the proposed scheme for different $T_{r}$ and network size.

The next interesting trend that has been observed in the graphs is the difference between total copies of the data packet that are forwarded by AHH-VBF is larger than the proposed scheme. It shows that the holding time difference between the potential forwarders is less than the propagation delay between those nodes. Hence, multiple nodes from the PFZ send copy of the same data packet, which results in more energy consumption that is discussed later in this section. In contrast, the proposed scheme computes holding time and exponentially scales the holding time using normalized energy factor to increase the holding time difference between potential forwarders. Consequently, it minimizes the data packet duplication and saves energy in the proposed scheme. The same performance metric has been investigated for different transmission ranges and fixed network size and a similar trend has been observed in Figure 6. On average, the proposed scheme generates 27.55% less copies of the data packet for all $T_{r}$ and network size of 450 nodes. Similarly, in $T_{r}=900$ m and all network size scenarios, the proposed scheme disseminates about 21.93% fewer copies of the data packet in the network. A detailed performance gain achieved by the proposed scheme in this regard is shown in Figure 7. The maximum performance gain achieved by the ESEVBF in contrast to the AHH-VBF is 30.72% for the network size of 450 nodes and $T_{r}=500$ m.

**Figure 8:**
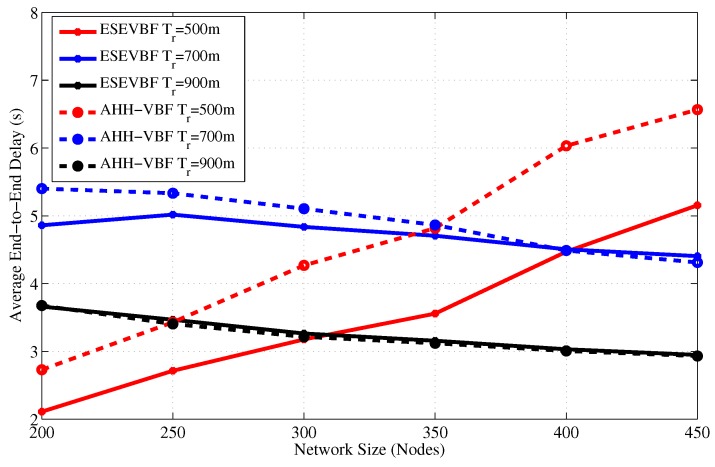
End-to-End delay between Source node that generated data message and Sink node versus the network size.

**Figure 9:**
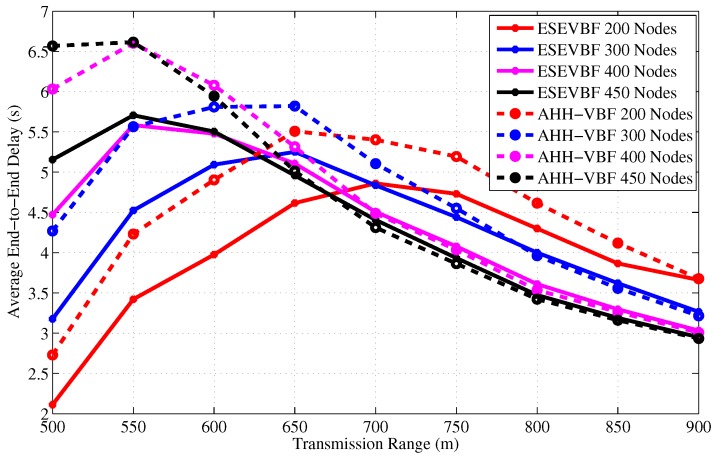
End-to-End delay between the source node that generated data message and Sink node versus transmission range.

Next, we investigate the end-to-end delay experienced by the successful data packets between the source *S* and sink node *D* for varying network size and transmission range as shown in Figure 8 and Figure 9, respectively. The overall delay experienced by a data packet includes processing, propagation, and the holding time delay at each forwarding stage in the network. The impact of network sparseness and denseness can be seen in those graphs. It is evident from the graphs that the end-to-end delay experienced by the AHH-VBF is larger than the proposed scheme. The main reason behind this behavior is our proposed potential forwarder selection algorithm. As stated earlier in the proposed forwarder selection algorithm section, all the nodes share their location and residual energy information with their 1-hop neighbors. When a node receives data packet from any one of its neighbors, it computes its own holding time. As we recall from our previous discussion that the holding time computation only requires the location plus depth information of sender node *S*, Sink node *D*, node’s own location, and the residual energy information. However, the same information of the neighboring nodes is also available in the neighborhood table of each node. Hence, the node can easily estimate the packet holding time of all common neighbors of *S* and node itself, which also fall in the *PFZ*.

It is also a well-established fact that computation energy cost is very small compared to communication and other operations of the node. Once the node estimates the holding time of its own as well as its neighbors, it checks whether its own holding time is smaller than the neighbors or not. If the receiving node’s holding time is smaller than its common neighbors, then instead of waiting for a long holding time duration, it forwards the packet after $\frac{HT_{p}^{i}}{2}$. However, the AHH-VBF does not exploit the neighborhood information available at the node and each node has to wait for a holding time duration before further relaying the data packet. This is the reason that despite the energy scaling and expansion of the proposed holding time, its end-to-end delay is smaller than the AHH-VBF. Subsequently, the same behavior can be observed for any network scenario, refer Figure 8 and Figure 9.

**Figure 10:**
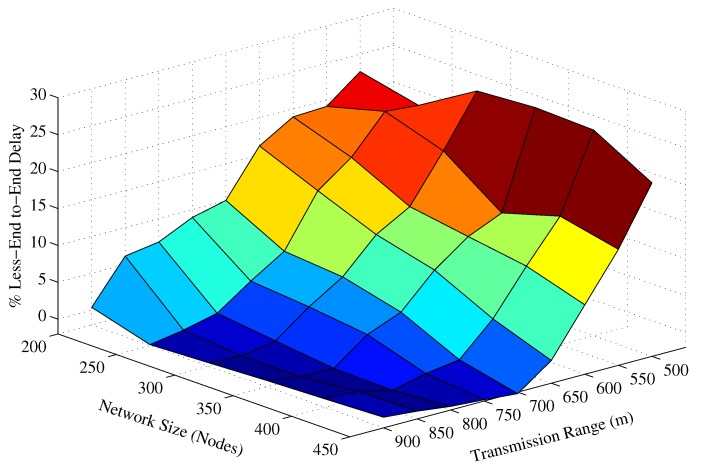
The overall percentage less End-to-End delay achieved by the proposed scheme for different $T_{r}$ and network size.

In Figure 8, the trend of the end-to-end delay for a small transmission range (e.g., $T_{r}=500$ m) is increasing with respect to the network size in contrast to the end-to-end delay behavior resulted by large transmission ranges. The main arguments behind this behavior are: (a) in case of a small $T_{r}$, the packet fails to reach *D* if it is relayed over multiple hops, due to channel impairments, path losses, high error rate of the acoustic channel, and so forth; (b) because of the network sparsity, end-to-end connectivity could not be established. Therefore, in a sparse network scenario, only the short hop-distant packets can reach *D* and experience a small end-to-end delay. On the contrary, in a dense network environment, the packet has to traverse a large number of hops, which causes a very long end-to-end delay. The similar trend is also observed in Figure 8. Furthermore, it is also noticed in Figure 8 that as $T_{r}$ increases, the end-to-end delay descends, which is due to the collision that leads to a large data packet loss. This collision happens when different potential forwarders relay the same data packet because of the small holding time difference and the large propagation delay between these forwarders, refer the theory related to Figure 2. Data packets in the proposed ESEVBF scheme experience about 23.77%, 12.6%, and 3.65% less end-to-end delay compared to AHH-VBF in any network size scenario with $T_{r}=500$ m, $T_{r}=600$ m and $T_{r}=700$ m, respectively. As the $T_{r}$ increases, the end-to-end delay of both the schemes becomes identical because the Data packet is forwarded through less number of hops and directly reaches the sink node. The overall performance gain (percent improvement in end-to-end delay) achieved by the proposed ESEVBF is shown in Figure 10.

**Figure 11:**
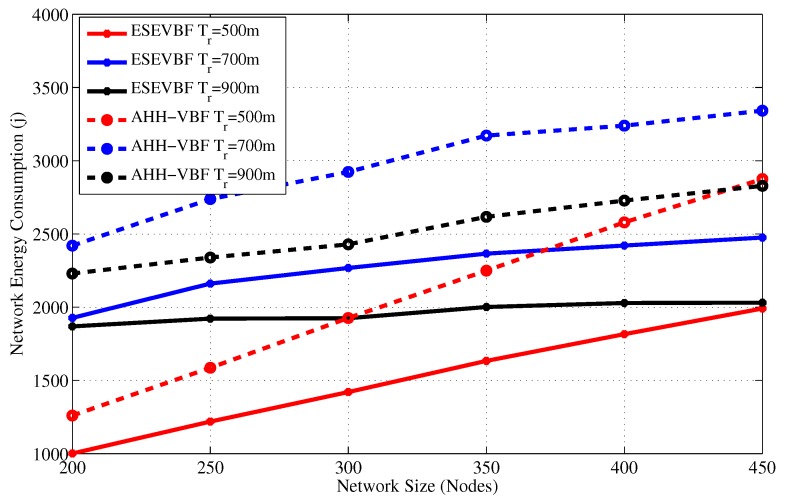
Overall network energy consumption versus the network size.

**Figure 12:**
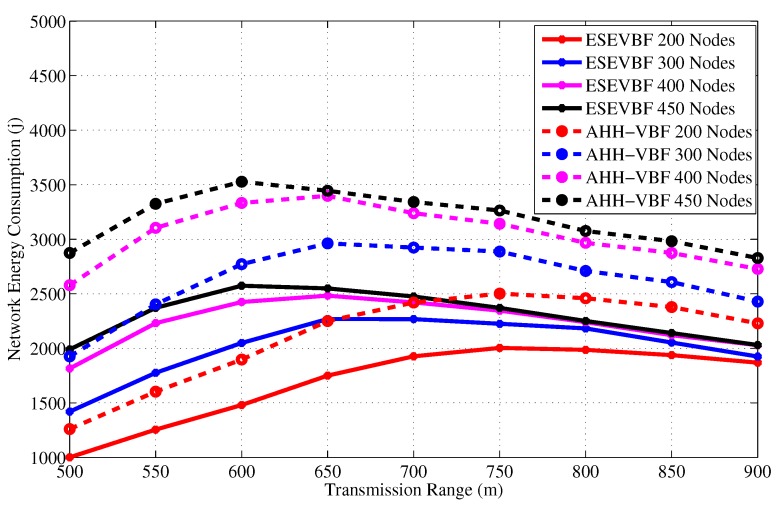
Overall network energy consumption versus the transmission range.

After the data packet broadcast and end-to-end delay analysis, we investigate the overall energy consumption in the network. Figure 11 and Figure 12 show the total energy consumption in the network during the whole simulation duration for different network size and transmission range, respectively. In underwater acoustic networks, transmission of a packet is the most energy consuming operation in the network compared to the packet reception, idle listening, sensing, and the processing operations. As data packets are large in size compared to the control packets, therefore, their contribution to the energy consumption dominates the energy consumed by the transmission of other packets or network operations. Therefore, the trends of the overall network energy consumption graphs are comparatively similar to the results that depict the total number of forwarded copies of the data packets in the network. Hence, the main reasons and arguments related to energy saving are similar to the one that avoids broadcast of more copies of the data packet. As a result, the overall energy consumption of the proposed scheme is less than the AHH-VBF.

**Figure 13:**
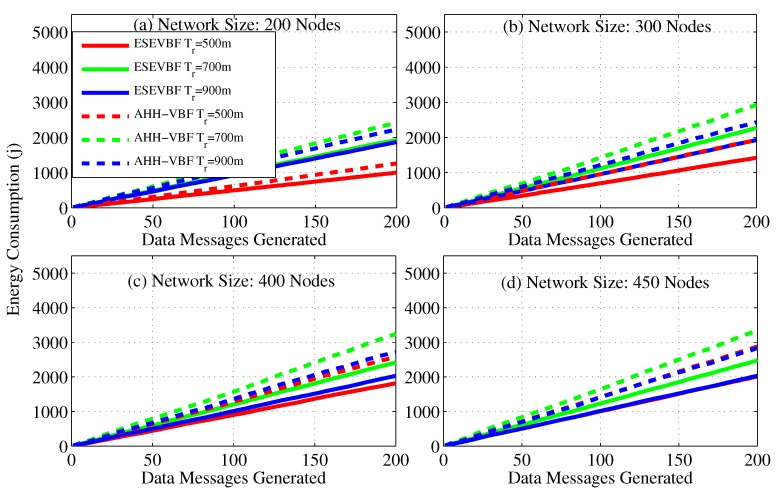
Overall network energy consumption for varying number of data packets generated by the source nodes in the network.

The simulation results show that the energy consumption in the sparse network is very small because most of the data packets could not be forwarded further in the upstream direction towards the Sink. Similarly, the number of forwarders, as well as the data packet receiving nodes (receiving energy consumption), are very few, which is one of the reasons for this small network energy consumption. Conversely, the opposite is the case for a dense network scenario where the successful communication of data packets increase energy consumption in the network. As the number of data sources are fixed in all the simulation scenarios, hence, we also recorded the overall network energy consumption after the individual broadcast of each data packet by the source node, refer Figure 13. The results show that maximum energy maximum energy saved by the proposed scheme is approximately 30.8%. The average percentage less energy consumed by the proposed scheme in comparison to AHH-VBF is summarized in Table 1.

**Figure 14:**
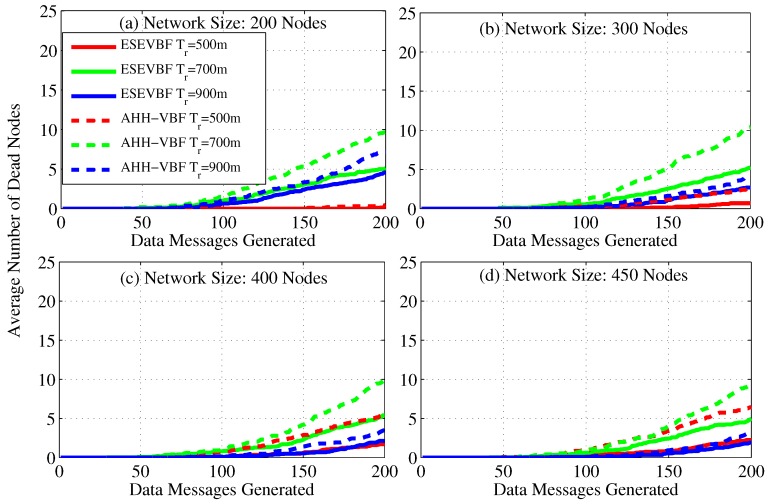
Number of dead nodes for varying number of data packets generated by the source nodes in the network.

As we already discussed that more energy is consumed by the underwater acoustic networks, which can deplete the battery power of some nodes (dead nodes) during the simulation duration. Therefore, we also studied the number of dead nodes during the simulation period, as shown in Figure 14. It is evident from the figure that the battery power of few nodes is completely consumed in the network scenario of 200 nodes and $T_{r}=500$ m, Figure 14a, because a small number of data traffic is handled by the network. In the similar network scenario but for the large $T_{r}$, more network nodes die out, because more data packets are communicated in the network. The battery power of more number of nodes deplete when the network becomes denser. However, it can easily be seen from the results that for $T_{R}=700$ m, the number of dead nodes is larger than the T=900 and 500 m, refer Figure 14a. The obvious phenomenon behind this is that in an extremely dense network scenario, a large number of potential forwarders get the chance to forward data messages because of a very small difference between their holding time. Therefore, the collision probability at the next hop increases and the next hop nodes fail to further communicate the data packet. Hence, the data packet communication is restricted only to a few hops in the network. On the other hand, in $T_{r}=700$ m, more data packets are successfully propagated in the network, which consumes more network energy and results in a large number of dead nodes in the network, refer Figure 14c,d for further details. Similarly, in the dense network scenario, e.g., Figure 14d, the small transmission range (e.g., $T_{r}=500$ m) consumes more energy due to the fact that most of the nodes participate in the Data packet forwarding. Hence, large number of nodes die out for small $T_{r}=500$ m compared to the large $T_{r}=900$ m. From the results, it can easily be seen that the battery power of a very small number of nodes is depleted during the simulation of ESEVBF. Resultantly, it increases the underwater monitoring duration and the network with ESEVBF can survive for a longer duration that the one using AHH-VBF.

**Figure 15:**
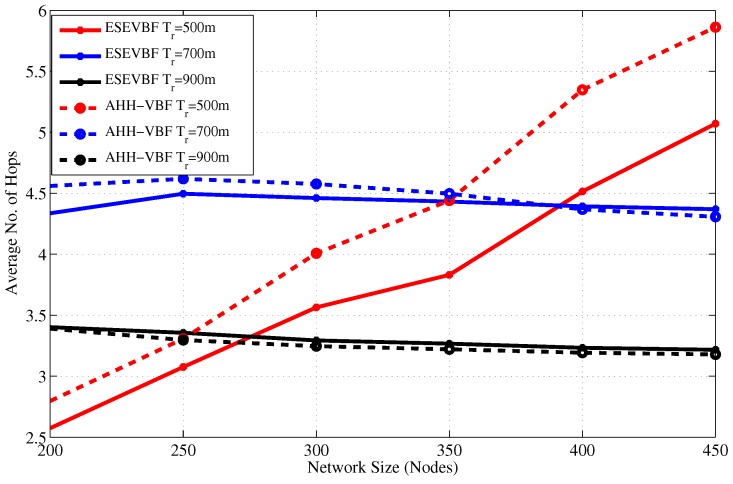
Average No. of Hops data messages traversed versus the network size.

**Figure 16:**
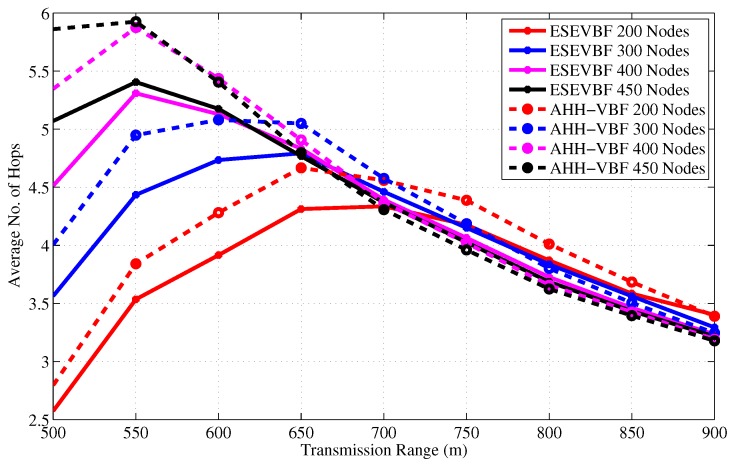
Average no. of hops data messages traversed versus transmission range.

From the above analysis, it is clear that the proposed scheme saves energy, reduces broadcast storm, and communicates data packets with less delay. In addition to these performance gains, the average number of hops that each data packet traverse from *S* to *D* is also analyzed for different network sizes and $T_{r}$, refer Figure 15 and Figure 16, respectively. The hop count data coincides with our previous analysis that for a small $T_{r}=500$ and network of 200 nodes, the average number of hops is smaller than the large $T_{r}$s. The rationale for this behavior is that the data messages from sources that are at distant location, fail to reach *D* due to unavailability of the path(s). However, when the network becomes dense, the data packet has to traverse many hops to reach *D* for $T_{r}=500$. On the other hand, it can easily be noticed from the results that the average number of hop counts of the proposed scheme is smaller than the AHH-VBF because the forwarder selection criteria mostly considers the node’s closeness towards the center of the virtual cylinder and the Sink node. Notwithstanding, the proposed scheme scales the holding time with residual energy factor plus the expanded ratio of the closeness towards the center of the virtual cylinder. This may increase the chances of other nodes to serve as potential forwarders that have large residual energy and slightly different virtual cylinder distance ratio. This is the reason that for large $T_{r}$, the average number of hops traversed by the Data packet in ESEVBF is slightly larger or almost identical to the AHH-VBF. The impact of this slightly larger hop count is reduced by taking the advantage of utilizing the neighborhood information in holding time calculation and reducing the holding time of the potential forwarder that has a smallest holding time among neighbors. Therefore, this marginally high hop count factor has not much impact in presence of the other significant performance gains achieved by the proposed scheme.

**Figure 17:**
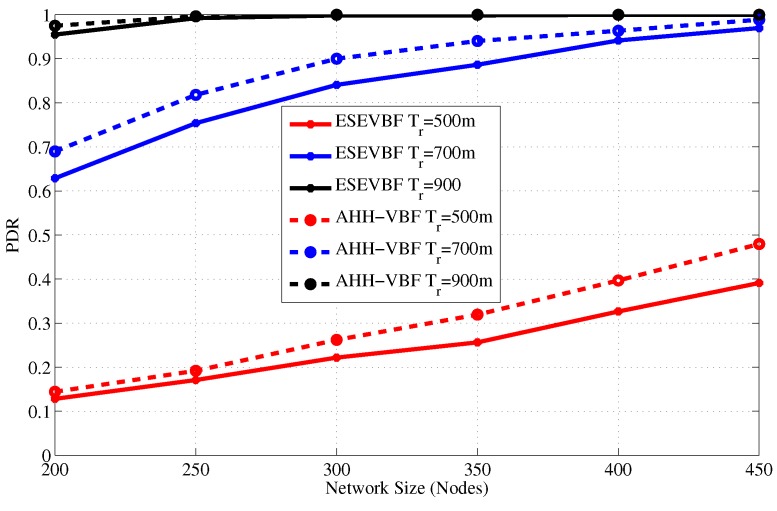
Average packet delivery ratio versus the network size.

**Figure 18:**
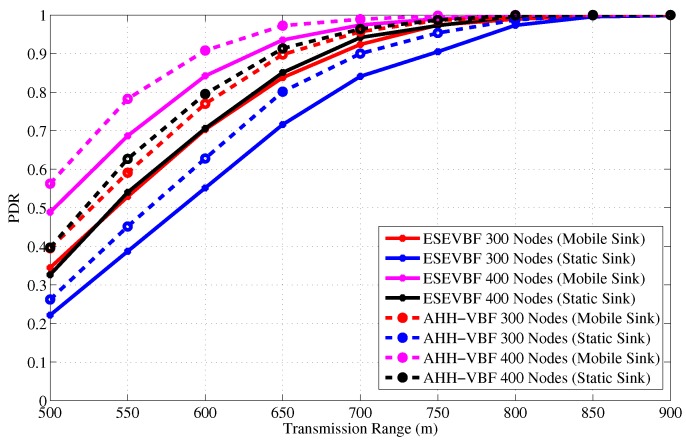
Average packet delivery ration versus the transmission range.

Along with those performance gains, finally, we simulated the packet delivery ratio or PDR $=\left(\frac{\mathrm{Successfully}\;\mathrm{received}\;\mathrm{data}\;\mathrm{at}\;D}{\mathrm{total}\;\mathrm{generated}\;\mathrm{data}\;\mathrm{by}\;S}\right)$. PDR is one of the fundamental performance measures of every routing strategy is also analyzed. Figure 17 and Figure 18 show the PDR comparison of the proposed and the AHH-VBF scheme for different network size and transmission range, respectively. Figures show that the proposed scheme has almost similar PDR as the AHH-VBF as the network size or $T_{r}$ is very large. On the other hand, the ESEVBF has slightly less PDR because ESEVBF selects the potential forwarders in the in PFZ that have closeness to the center of the cylinder and have larger residual energy. However, due to small number of nodes in the PFZ due to network sparseness, it is quite difficult to find suitable forwarders. Hence, the PDR of the ESEVBF is lower in those network scenarios. Conversely, in the dense network scenario, the chances of finding the suitable forwarder from the PFZ becomes very high that increases the PDR. The above results show that the proposed scheme achieves energy efficiency, broadcast less number of Data packets, and has lower end-to-end delay at the cost of slightly lower or almost identical PDR is different network scenarios.

In the next subsection, we briefly analyze and contrast the impact of Sink mobility on the performance gain of both ESEVBF and the AHH-VBF schemes.

### 4.3. Simulation Results in the Mobile Sink Scenario

In the previous section, we critically analyzed and reasoned about the performance gain of the ESEVBF in the network scenario with the static Sink. However, the literature suggests that introduction of the Sink mobility enhances the network performance in terms of small end-to-end delay, reduces the broadcast of the number of Data packets, minimizes energy consumption and increases the network lifetime, and so on. Although the AHH-VBF has not been tested in the mobile Sink network scenario, however, we extensively simulated AHH-VBF as well as our proposed ESEVBF in the mobile Sink paradigm and the results are discussed below.

**Figure 19:**
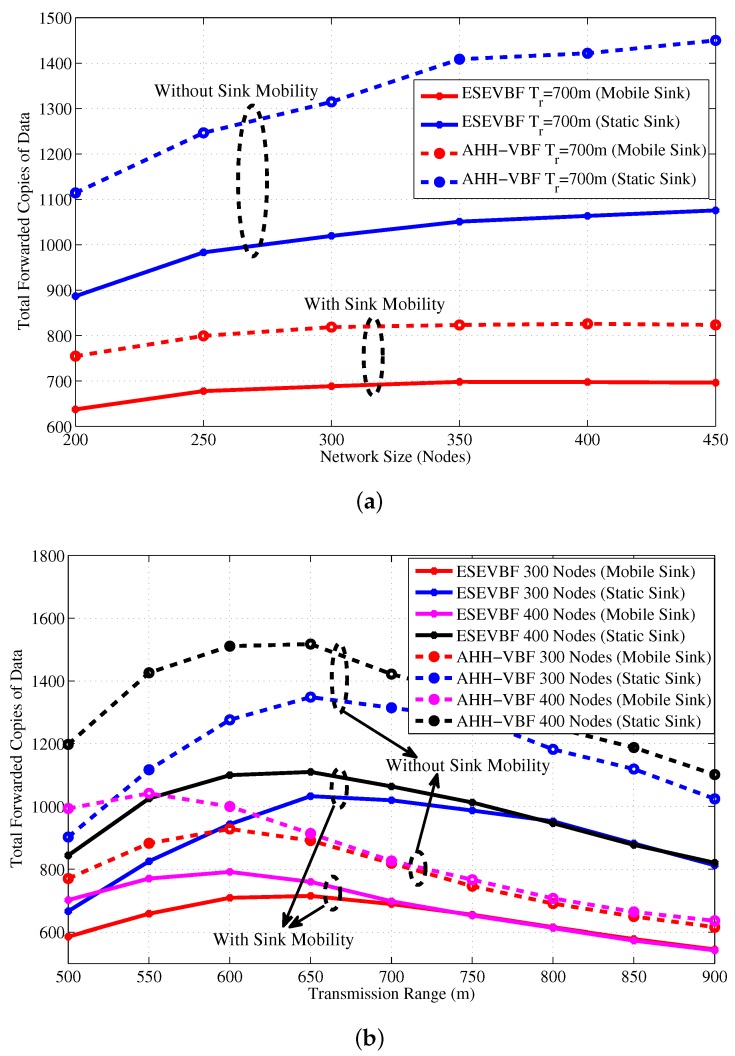
Total copies of data message forwarded in the network with and without Sink mobilty for different (**a**) network size (**b**) transmission range.

**Figure 20:**
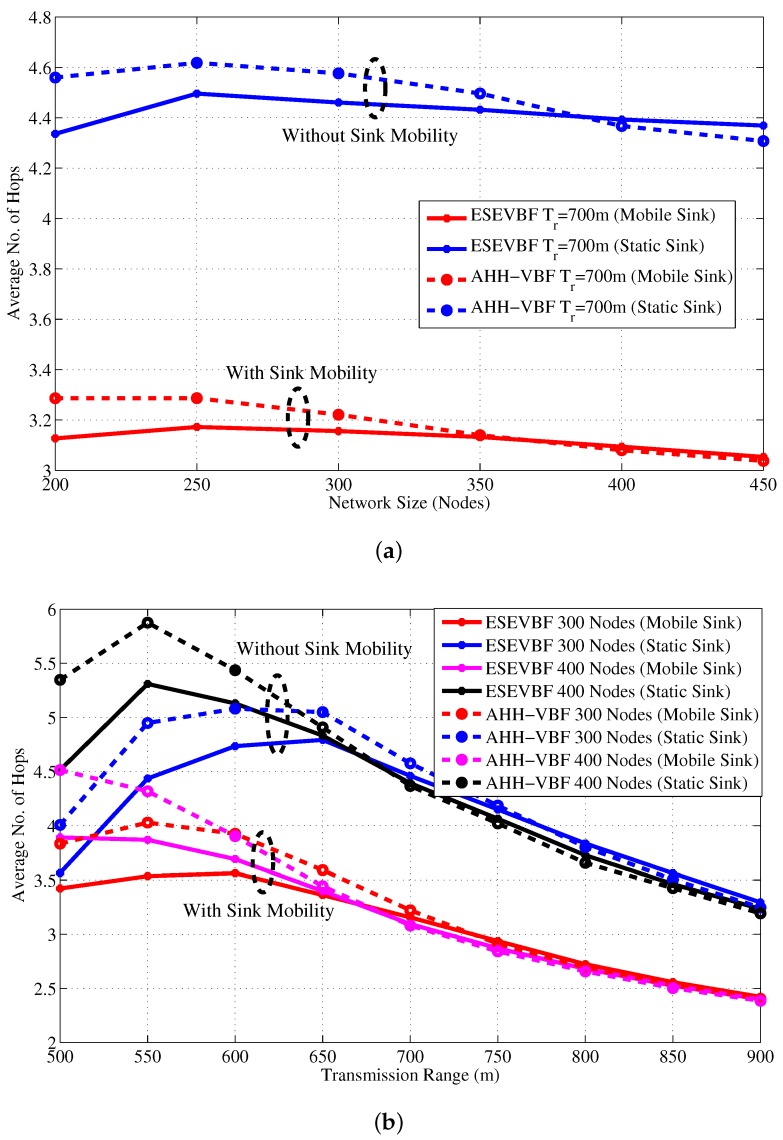
Average number of hops the data message needs to traverse in network to reach static and mobile Sink (**a**) Network with varying size (**b**) Network with varying transmission range.

Figure 19 shows the total number of copies processed within the network of varying size and transmission range. It is evident from the figure that the Sink mobility minimizes the broadcast of the number of data packet copies in the network. The reason behind this phenomenon is that Sink moves within the network and passes near the data generating and forwarding nodes. Hence, these nodes just have to forward data packet at fewer hops to reach the Sink that is within the close proximity of these nodes, refer Figure 20 that shows average number of hops the data packet traverses in the network to reach the Sink. Figure 19a,b explicitly show that the ESEVBF and AHH-VBF forward far more less copies of data packet in the mobile Sink scenario as compared to the one with static Sink. The analysis shows that ESEVBF for any network size in a mobile Sink scenario processes 12.6%, 24.4%, 32.5%,⋯, and 34.0% less number of data messages in the network compared to static Sink scenario for $T_{r}$ of 500 m, 600 m, 700 m,⋯, and 900 m, respectively. Similar data packet gain is also achieved by the AHH-VBF in the network with Sink mobility. Additionally, it is also obvious from the figure that ESEVBF outperforms the AHH-VBF in any network scenario. The mobility enables the Sink to vary its proximity respective to the data packet source and the forwarder nodes. Hence, the number of hops traversed by the data packet is far more less in the mobile Sink network than the static Sink network scenario, refer Figure 20.

**Figure 21:**
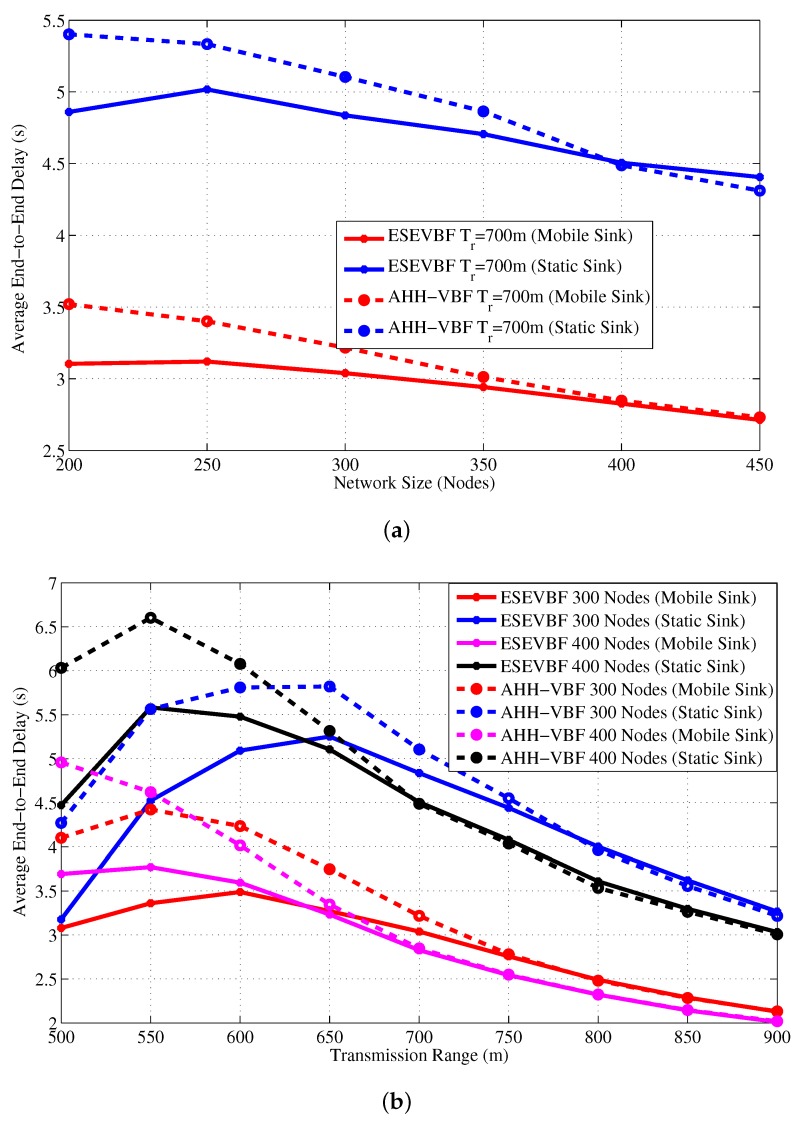
End-to-end delay experienced by the data message in a network with static and mobile Sink for varying (**a**) network size (**b**) transmission range.

Above analysis shows that the Sink mobility in the underwater network scenario significantly reduces the number of data message copies and the number hops the message traverses in the network. In result, it can easily be predicted that the end-to-end delay must also be alleviated in this network setting. Figure 21 shows the end-to-end delay experienced by the data message in a network with static and mobile Sink for varying (a) network size (b) transmission range. A notable difference in the end-to-end delay can be observed in the figure. On average, the ESEVBF lessens about 28.5%, 31.0%, 31.4%,⋯, and 34.0% of end-to-end delay in the network with mobile sink than the network without sink mobility for the size of 200, 250, 300,⋯, and 450 nodes and all $T_{r}$. Notwithstanding, the AHH-VBF achieves 28.3%, 29.0%, 29.9%,⋯, and 33.1% less end-to-end delay for the network of 200, 250, 300,⋯, and 450 nodes, which is closer to ESEVBF’s performance gain.

**Figure 22:**
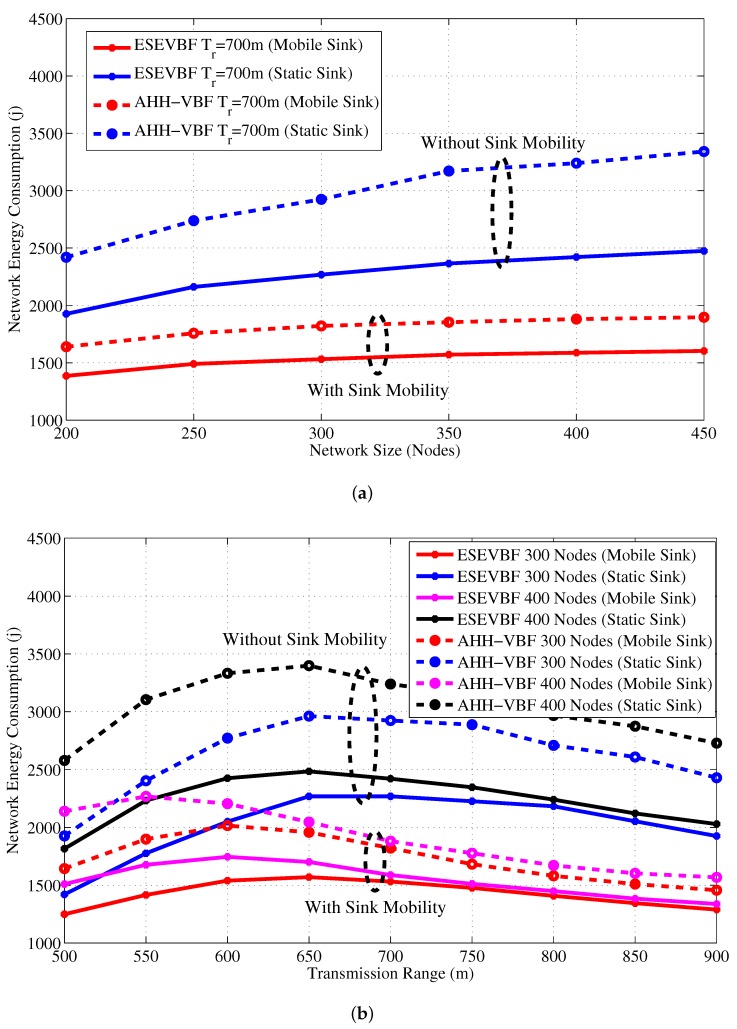
Network energy consumption in the static and mobile Sink network scenario for varying (**a**) network size (**b**) transmission range.

**Figure 23:**
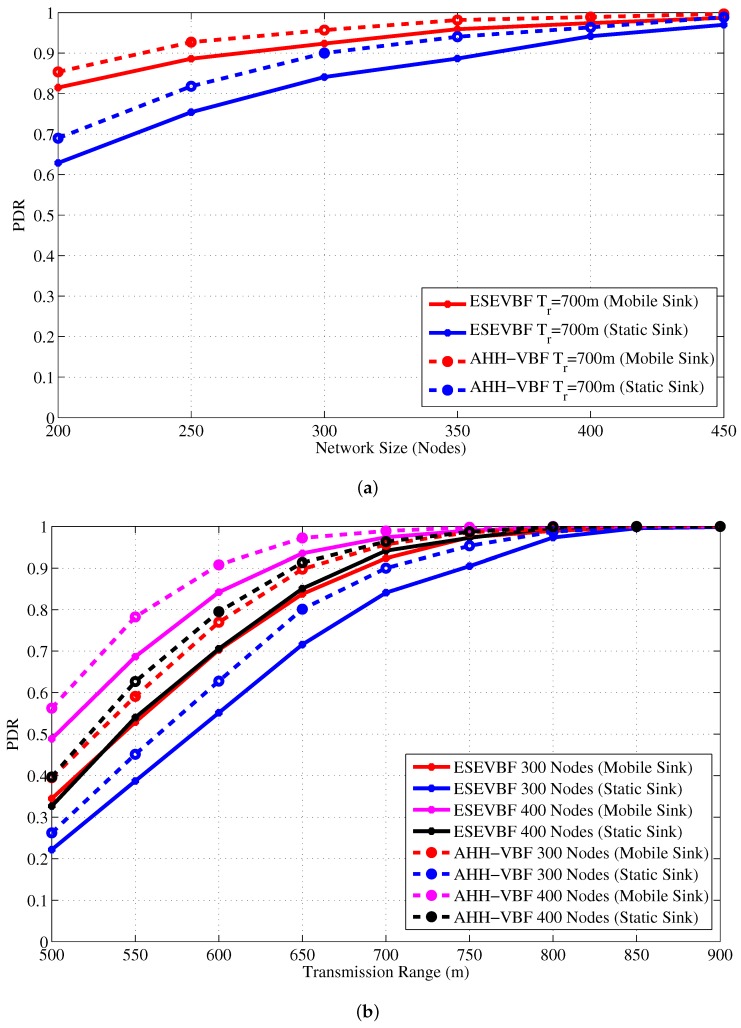
PDR in the static and mobile Sink network scenario for varying (**a**) network size (**b**) transmission range.

**Figure 24:**
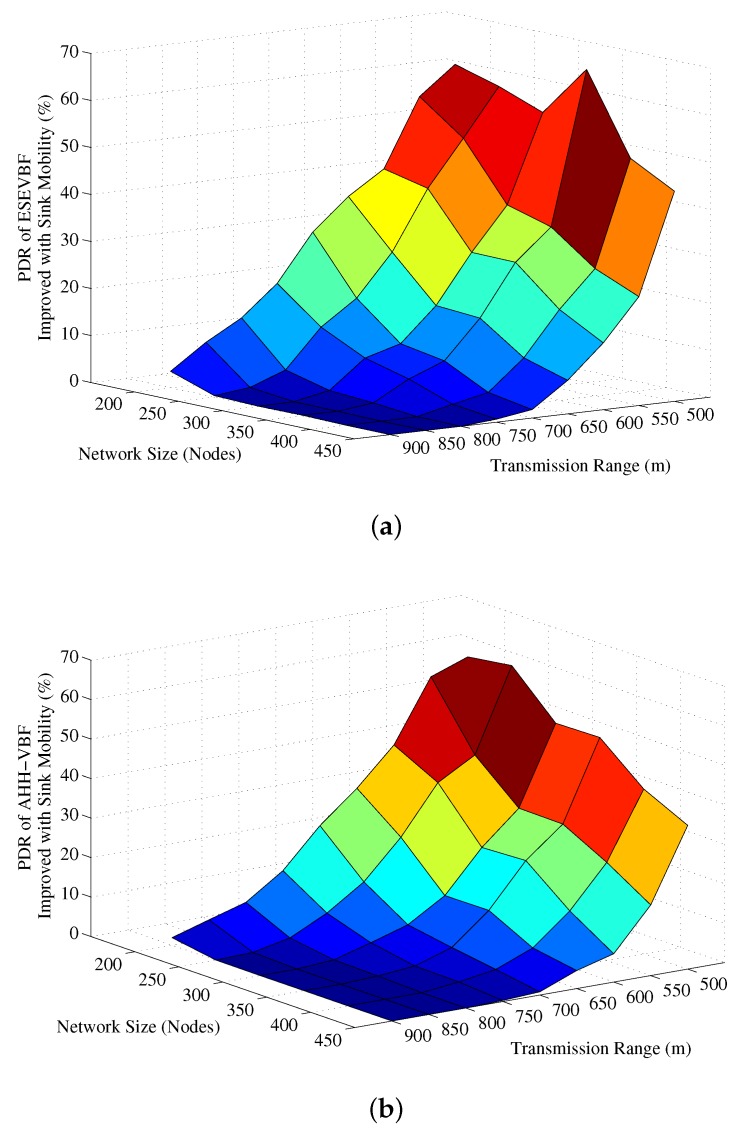
PDR alleviated after the introduction of the mobile Sink in (**a**) ESEVBF (**b**) AHHVBG.

Next, the influence of Sink mobility on the overall network energy consumption is shown in Figure 22. Results in that figure show the similar impact on the energy conservation as it has on the data copies processed in the network. It also verifies the claims in the literature that Sink mobility achieves energy efficiency in the network, which is about 36% more energy conserved than the static Sink scenario. Finally, the PDR improved by the proposed and conventional scheme is shown in Figure 23. The overall PDR improved by the Sink mobility for ESEVBF and AHH-VBF Figure 24a,b, respectively. From the above discussion, we can easily conclude that the Sink mobility improves the performance of the routing protocols.

## 5. Conclusions

An energy scaled and expanded vector based routing (ESEVBR) scheme is proposed in this paper. ESEVBR provides energy fairness and reduces the packet broadcast by scaling and expanding the holding time with the residual energy and ratio of projection distance to vector and width of the virtual pipeline. The new holding time signifies the difference between holding times of the potential forwarders, which reduces the data packet broadcast and maintains the energy fairness in the network. The simulation results show that the proposed ESEVBR is approximately 5% more energy efficient, experiences 14.6% less delay, and generates about 5% fewer data packets, compared to AHH-VBF. However, ESEVBR maintains similar PDR and very less number of nodes die in the network when ESEVBR is used as a forwarding technique in the underwater sensor network.

## Figures and Tables

**Table 1:** Overall energy saved by the proposed scheme compared to AHH-VBF.

Network Size	200	250	300	350	400	450
$T_{r}=500$ m	20.6	23.1	26.3	27.4	29.6	30.8
550 m	21.7	24.1	26.1	25.9	28.1	28.7
600 m	21.9	23.6	26.0	26.9	27.2	27.0
650 m	22.2	21.9	23.4	25.4	26.9	26.0
700 m	20.4	21.1	22.4	25.4	25.3	25.9
750 m	19.9	18.7	22.9	24.7	25.3	27.4
800 m	19.3	16.6	19.5	21.9	24.5	26.8
850 m	18.6	17.9	21.3	23.6	26.2	28.2
900 m	16.2	17.8	20.8	23.5	25.6	28.2
**Energy Saved**	**20.1%**	**20.5%**	**23.2%**	**25.0%**	**26.5%**	**27.7%**
